# Trends and Patterns for the Use of Herbal Medicinal Products for Gynaecological Ailments

**DOI:** 10.1002/ptr.70321

**Published:** 2026-04-06

**Authors:** Alexandra Drebka, Annika J. Scholl, Teresa Ochs, Olaf Kelber, Ralph Mösges, Beatrice E. Bachmeier

**Affiliations:** ^1^ Institute of Pharmaceutical Biology, Goethe‐University Frankfurt am Main Germany; ^2^ R&D, Phytomedicines Supply and Development Center, Bayer Consumer Health Steigerwald Arzneimittelwerk GmbH Darmstadt Germany; ^3^ Kooperation Phytopharmaka GbR Bonn Germany; ^4^ Institute of Medical Statistics and Computational Biology, University of Cologne Cologne Germany; ^5^ ClinCompetence Cologne GmbH Cologne Germany

**Keywords:** drug utilisation, gynaecological complaints, herbal drugs, herbal medicinal products, patient‐reported outcomes, trends and patterns

## Abstract

Most Germans consider herbal medicinal products (HMPs) to be an important supplement to conventional medicine. Despite existing clinical evidence for safety and efficacy, they are still not sufficiently integrated into drug therapy of gynaecological complaints in everyday practice. By analysing patient‐reported outcomes (PROs), this gap in medical care can be closed. Real‐world data was extracted from the pharmaco‐epidemiological database PhytoVIS. We analysed a sample (*n* = 1658) containing PROs from women who utilised HMPs to treat their gynaecological complaints applying descriptive and non‐parametric bivariate statistical tests. Perceived effectiveness and tolerability of HMPs was rated as very good. For the treatment of menstrual complaints, *Vitex agnus‐castus* L. was primarily used, and *Actaea racemosa* L. for menopausal complaints. Various herbal drugs were applied for uncomplicated urinary tract infections (uUTIs), but mainly *Arctostaphylos uva‐ursi* (L.) Spreng. Regarding the pharmaceutical form, herbal teas were preferred for the treatment of uUTIs or by very young or elderly women. All other pharmaceutical forms were favoured for menstrual and menopausal complaints or middle‐aged women. The pharmaceutical form did not impact the perceived therapeutic effectiveness. Our results provide valuable insights into patient preferences and show options for their integration into existing treatment strategies. By identifying the most popular and efficacious plants for certain gynaecological ailments, we support healthcare providers to better address the growing demand for complementary treatment options. This knowledge helps in tailoring healthcare to meet patient needs and to ensure the safety and efficacy of HMPs.

**List of Abbreviations**
EMA: European Medicines Agency
HMPC: Committee on Herbal Medicinal Products
HMP: herbal medicinal product
HMPs‐eT: herbal medicinal products except teas
HTs: herbal teas
Mens: menstrual complaints
Meno: menopausal complaints
uUTIs: uncomplicated urinary tract infections
PRO: patient‐reported outcomes

## 1 Background

A representative survey recently conducted in Germany revealed that two‐thirds of the participants consider herbal medicinal products (HMPs) to be an important supplement to conventional medicine (Compafarm 2021). Dissatisfaction with non‐herbal medicines, positive experiences with HMPs and their natural origin, as well as traditions and family lore are the main reasons to prefer HMPs (Welz et al. 2018, 2019b).

Recent pharmaco‐epidemiologic research reveals that HMPs are utilised predominantly for the treatment of mild to moderate complaints and for acute as well as chronic ailments (Wegener et al. 2021). Particularly, women are more likely than average (79%) to believe in their pharmacological effects and value their good tolerability (Compafarm 2021).

In this context the interest in HMPs for the treatment of non‐life‐threatening gynaecological ailments like menstrual and menopausal complaints, as well as uncomplicated urinary tract infections (uUTIs) is rising.

However, gynaecologists in everyday practice like to prescribe contraceptives for menstrual complaints—which is actual equivalent to an off‐label use—although more and more women in Germany are dissatisfied with the prescription of hormone preparations. The rising awareness of women not to use contraceptives to treat their menstrual problems because they suppress the body's own hormone production (Behrens 2024) and can cause serious side effects, such as venous thrombosis (Khialani et al. 2020), is reflected in the declining number of prescriptions reported by one of the biggest German statutory health insurance companies (AOK, dt.: Allgemeine Ortskrankenkasse). Accordingly, the proportion of young women under 22 years of age taking contraceptives has fallen from around 46% in 2010 to 25% in 2023 (Behrens 2024).

Treating menstrual pain with NSAIDs is also viewed critically by young women because it can cause undesirable gastrointestinal or adverse neurological effects (Marjoribanks et al. 2015).

Hormone preparations for menopausal symptoms have also become increasingly unpopular since the WISDOM study pointed out that hormone therapy increases the risks for breast cancer and cardiovascular events (Vickers, Martin, and Meade 2007; Vickers, MacLennan, et al. 2007). In reaction to these results, the prescriptions for oestrogen or oestrogen–progestogen combination preparations have been declining (RKI 2008) and the German S3‐guidelines recommend hormonal therapy only for short‐term treatment, in low doses, and the decision about its use should be made on an individual basis (DGGG L 2020).

For the treatment of uUTIs, antibiotics are often prescribed although a clear diagnosis of the causing pathogen has not been made (Kranz et al. 2017). This represents a major problem for the healthcare system (Kranz et al. 2017; Rothberg and Wong 2004; Wagenlehner et al. 2013), because antibiotics can damage the patient's microbiome ((Hrsg.) DGfUeV 2024) and subsequently lead to fungal infections of the vagina (Jeannin and Meier 2018).

The three gynaecological complaints mentioned above can be regarded as non‐life‐threatening diseases—at least as long as they do not lead to complications. It is therefore conceivable to treat them with HMPs, which are an important part of self‐mediation in Germany. HMPs are defined as any medicinal product, exclusively containing as active ingredients one or more herbal substances, one or more herbal preparations, or a combination of the two (as defined by the European Medicines Agency—EMA) (HMPC 2018a). HMPs are available as herbal teas (HTs) or various other pharmaceutical dosage forms (e.g., tablets, syrup, drops, capsules).

For the treatment of menstrual complaints, the HMPC (Committee on Herbal Medicinal Products) of the EMA recommends a monographed herbal extract of *Vitex agnus‐castus* L. (fruits, well‐established use) (HMPC 2018b). Their assessment is based on results from clinical studies which revealed that *Vitex agnus‐castus* L. is superior to placebo for the treatment of menstruation‐related symptoms (Schellenberg 2001; Schellenberg et al. 2012; He et al. 2009).

Additionally, the HMPC recommends extracts of *Actaea racemosa* L. (rhizome, well‐established use) (HMPC 2018c), for the treatment of menopausal symptoms, especially hot flashes and excessive sweating (Briese et al. 2007; Drewe et al. 2013; Mohammad‐Alizadeh‐Charandabi et al. 2013; Bai et al. 2007; Osmers et al. 2005).

Where appropriate, uUTIs can also be treated with HMPs like for example, the diuretic *Equisetum arvense* L. (herba), *Betula pendula* Roth (leaves), or *Urtica urens* L. (leaves) (Engelsing 2016). *Arctostaphylos uva‐ursi* (L.) Spreng. (leaves) is used due to its antibacterial effects and *Solidago virgaurea* L. (herba), *Matricaria chamomilla* L. (flora), and *Tropaeolum majus* L. (herba) have an anti‐inflammatory and disinfectant effect (Engelsing 2016; Wichtl et al. 2016). In approximately 85% of the cases of a RCT, a combination of *Levisticum officinale* W.D.J. Koch (roots), *Salvia rosmarinus* Spenn. (leaves) and *Centaurium erythraea* Rafn (herba) was not inferior to the standard antibiotic therapy, fosfomycin, which is one of our ‘reserve antibiotics’, and at the same time caused fewer gastrointestinal side effects (Wagenlehner et al. 2018). The list of references given here shows that there is already good clinical evidence that justifies the use of HMPs for the mentioned ailments; however, they are still underrepresented in treatment strategies.

In order to substantiate the already existing clinical results, we analysed a subset of real‐world data taken from the pharmaco‐epidemiological database PhytoVIS which contains indication‐related information from patients on the therapeutic use of HMPs (Wegener et al. 2021). Our sample contained 1658 cases from everyday practice of women who used herbal preparations to treat their gynaecological complaints. The dataset provides valuable information from everyday practice which helps to understand (i) which HMPs are preferred to treat certain medical conditions, (ii) how HMPs are applied in self‐medication (regularly or when symptoms appear), (iii) if there are favoured forms of administration (e.g., herbal teas or herbal medicines), (iv) if the severity of the symptoms impacts patients' choice of HMPs and (v) how patients perceived the therapeutic effectiveness and undesired effects of HMPs.

Together with already existing clinical evidence, this information helps to tailor healthcare to meet the patients' rising demand for HMPs.

The aim is to display how women with gynaecological complaints integrate HMPs in their treatment strategies and how they perceive their effectiveness and tolerability.

## 2 Materials and Methods

### 2.1 Data Source

This is a retrospective study of real‐world data consisting of patient‐reported outcomes (PROs) collected between 2014 and 2016.

The study sample was taken from the pharmaco‐epidemiological database PhytoVIS established in 2013 by the German scientific association ‘Kooperation Phytopharmaka’ and the Institute for Medical Statistics, Informatics and Epidemiology (now: Institute of Medical Statistics and Computational Biology, IMSB) of the University of Cologne. The anonymised data contains cross‐product and indication‐related information on the use of HMPs for the treatment of a variety of ailments, as previously described (Wegener et al. 2021). Data collection for the PhytoVIS study complies with the European Network of Centres for Pharmacoepidemiology and Pharmacovigilance (ENCePP) criteria on pharmacological studies.

For being included into the PhytoVIS study, the participants had to have experiences with the intake of herbal drugs within at least eight weeks prior to the interview and they had to give their consent to be interviewed. For data collection, thoroughly trained pharmacy and medical students have conducted anonymous interviews in pharmacies and general practices regarding personal experiences with the therapeutic use of HMPs. Therefore a questionnaire designed by the ‘Kooperation Phytopharmaka’ was used, consisting of 20 items including information on complaints/diseases, product information, drug use, concomitant factors/diseases and demographic data.

### 2.2 Ethics

The Ethics Commission of the University Hospital of Cologne approved the project (reference: 14‐101). The ethical standards of the institutional and/or national research committee and the 1964 Helsinki declaration and its later amendments or comparable ethical standards involving human participants were met. The study protocol of PhytoVIS is registered in the European Network of Centres for Pharmacoepidemiology and Pharmacovigilance (ENCePP), according to the guidelines of the ENCePP Guide on Methodological Standards in Pharmacoepidemiology (EMA/95098/2010, amended). The study is identified by the ID number 28113 and has the corresponding EU PAS number EUPAS7082.

### 2.3 Variables

The PhytoVIS questionnaire consists of a total of 20 questions relating to complaints, product information, accompanying factors or concomitant diseases and demographic data which have been transferred into 103 variables.

The following variables were chosen for our study:

− 1 variable indicating the type of ailment
− 1 variable indicating the age in predefined categories (12–17 years, 18–30 years, 31–50 years, 51–65 years, 66–75 years)
− 3 variables referring to the applied HMP from which we generated 2 variables referring to the plant extract defined as either mono‐preparation or combi‐preparation
− 1 variable indicating the pharmaceutical form from which we generated two variables regarding HMPs except herbal tea or herbal tea
− 1 variable referring to the treatment habit (‘daily’ or ‘as required’)
− 1 variable regarding the duration of use from which we generate 1 variable categorising the duration in ‘short‐term use (≤ 14 days)’ and ‘long‐term use (> 14 days)’
− 1 variable regarding the severity of the symptoms (six‐grade Likert Scale ranging from 0 ‘no complaints’ to 5 ‘strongest complaints’)
− 1 variable regarding the perceived effectiveness of the HMP (‘very good’, ‘moderate‐distinct’, ‘minimal‐mild’, ‘unchanged‐worsened’)
− 1 variable regarding the perceived undesired effect from the application of the HMP (‘no’, ‘no impairment’, ‘significant impairment’, ‘outweigh effectiveness’)
− 1 variable regarding the perceived specified undesired effects

### 2.4 Study Cohort

**FIGURE 1:**
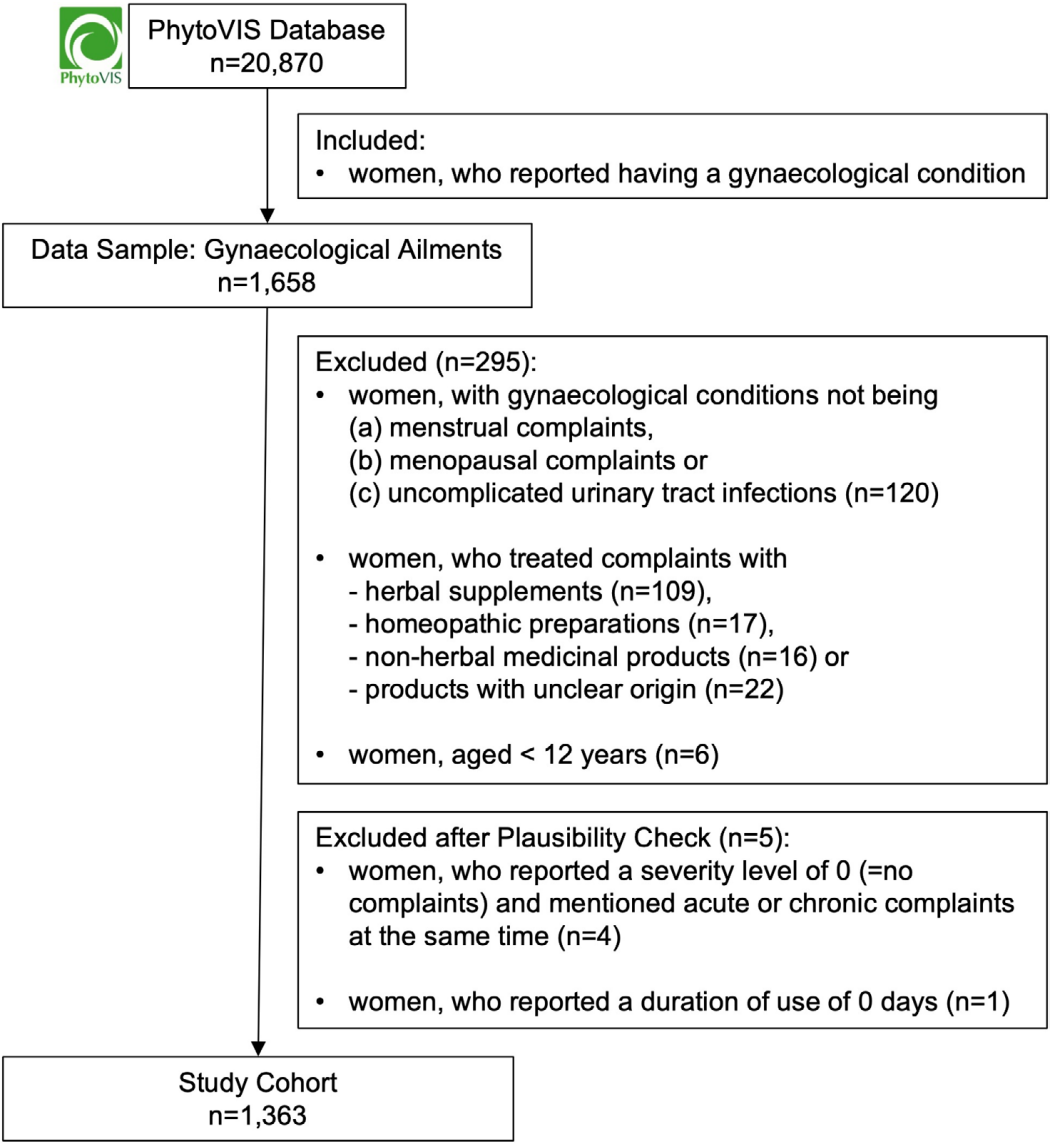
Selection of our PhytoVIS study population according to defined inclusion and exclusion criteria. After the selection process 1363 data entries remained.

From the PhytoVIS data set, which originally contained 20,870 patients, we selected our study population according to defined inclusion and exclusion criteria (Figure 1):

Inclusion:

− Women with gynaecological complaints who reported to treat their symptoms with HMPs (*n* = 1658).

Exclusion:

− Age < 12 years (*n* = 6).
− All conditions other than menstrual complaints, menopausal complaints and uncomplicated UTIs (*n* = 120).
− Preparations other than HMPs: herbal supplements (*n* = 109), homeopathic preparations (*n* = 17), non‐herbal medicines (*n* = 16), preparations of uncertain origin (*n* = 17).

Exclusion after plausibility check:

− Have no symptoms at all (severity level = 0) and at the same time mention acute or chronic symptoms (*n* = 4).
− Use HMPs for 0 days (duration of use = 0 days) (*n* = 1).

### 2.5 Grouping of the Study Cohort

The study cohort was grouped into the three indications menstrual complaints, menopausal complaints, and uUTIs according to the following criteria:

− Women provided information about the type of their ailment;
− Age (age group of the participant);
− Applied HMP.

We applied a combination of all three criteria in order to allocate every single data entry to one of the three ailments. According to this allocation process, 222 data entries were assigned to menstrual complaints, 301 to menopausal complaints, and 840 to uUTIs.

### 2.6 Statistical Analysis

Descriptive statistics were applied to analyse the data regarding frequency and percentage. Non‐parametric bivariate statistical tests (Mann–Whitney *U* test and chi‐squared test) were applied where appropriate. Statistical significance was at *α* = 0.05. All statistical analyses were performed using IBM SPSS Statistics for Windows (version 29.0, IBM Corp. Armonk, NY, USA). GraphPad Prism (version 10.03.275, GraphPad Software LLC, Boston, USA) was used for illustrating the results.

## 3 Results

### 3.1 Preferences for Certain Pharmaceutical Forms

In general, the women in our dataset applied both ‘herbal medicinal products except teas’ (HMPs‐eT) as well as herbal teas (HTs) to treat their gynaecological complaints with distinct differences concerning the type of ailment and age.

### 3.2 Type of Ailment

**FIGURE 2:**
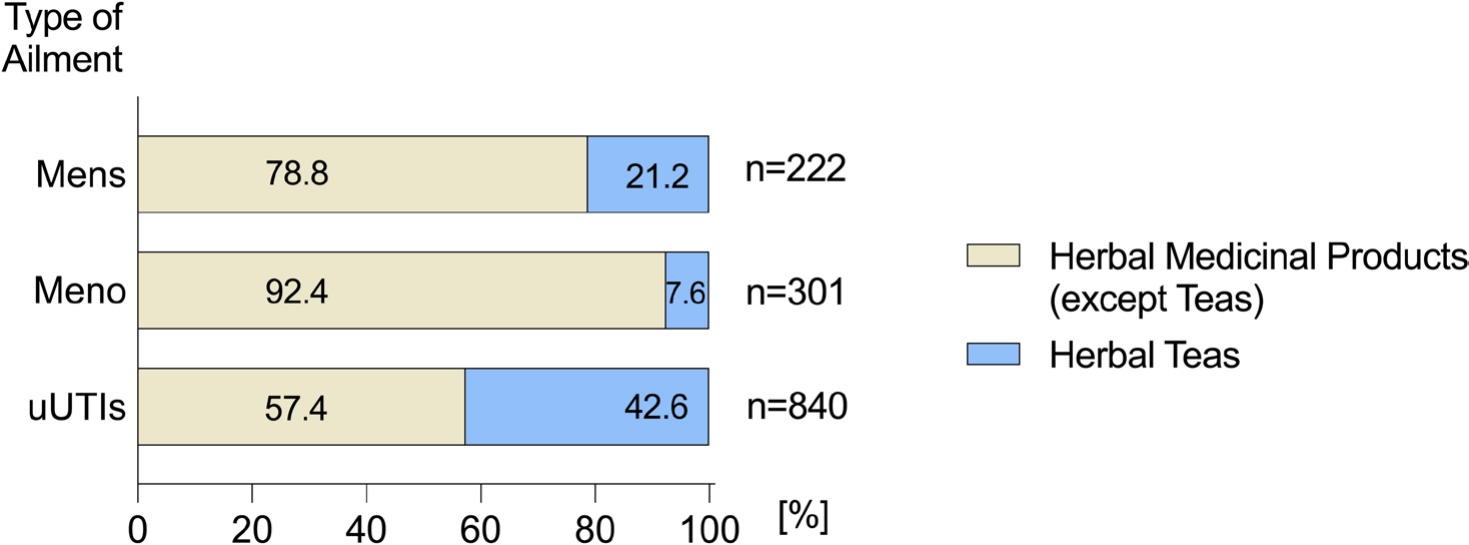
Preferences for herbal medicinal products (except Teas; HMPs‐eT) or herbal teas (HTs) in relation to type of ailment—For the treatment of menstrual (Mens, *n* = 222) and menopausal complaints (Meno, *n* = 301) women predominantly preferred HMPs‐eT. For uncomplicated urinary tract infections (uUTIs, *n* = 840) the utilisation of HTs was more pronounced compared to the other two ailments.

The majority of women with menopausal complaints preferred the use of HMPs‐eT (92%), followed by menstrual complaints (79%) and uUTIs (only 57%). The trend to apply preferably HTs was highest for the treatment of uUTIs (43%) compared to menstrual (21%) or menopausal complaints (only 7%) (Figure 2).

### 3.3 Age

**FIGURE 3:**
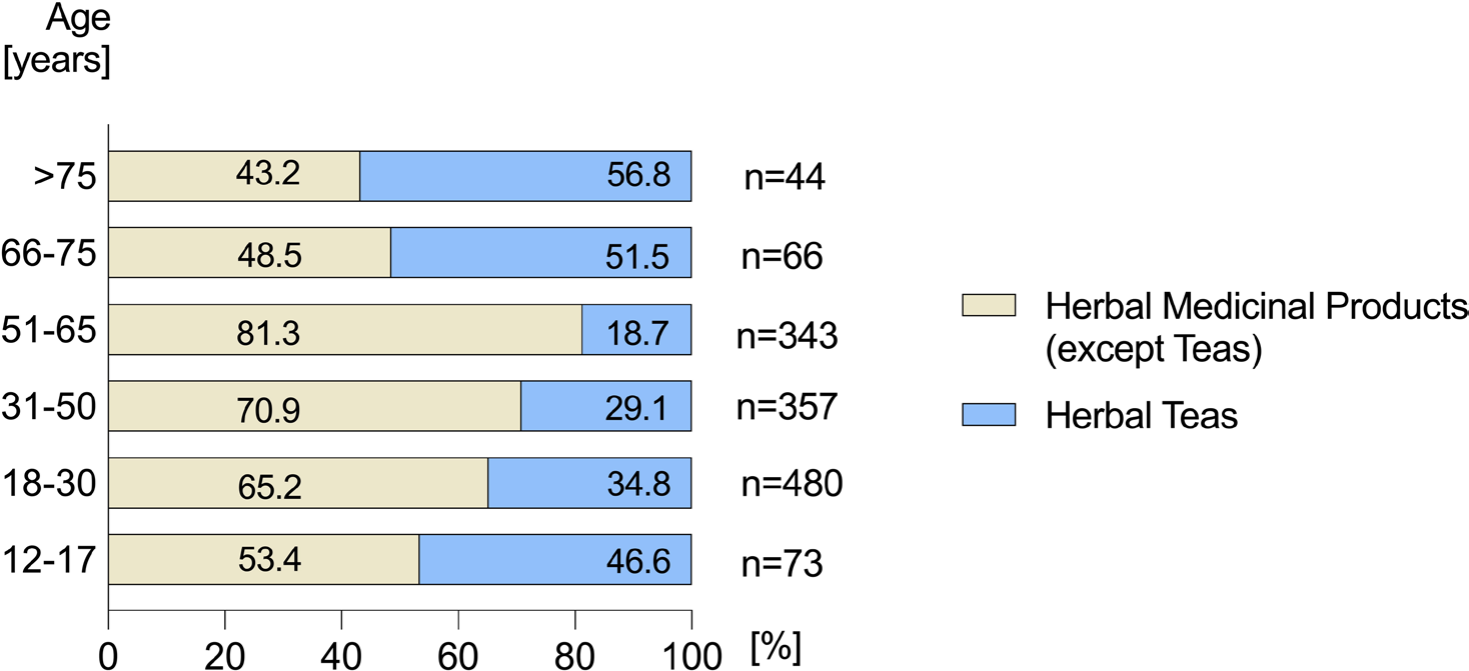
Preferences for herbal medicinal products except Teas (HMPs‐eT) or herbal teas (HTs) in relation to age—A clear preference for HMPs‐eT was observed in women aged 18–65 years.

Regardless of the type of ailment, the utilisation of HMPs‐eT in comparison to HTs was highest by women in the age group 51–65 years (81%), followed by 31–50 years old (71%). In the age groups of teenagers (47%) and elderly women starting at the age of 66 years (51% and 57%), a trend towards applying HTs is visible (Figure 3).

### 3.4 Combined View on Type of Ailment and Age

**FIGURE 4:**
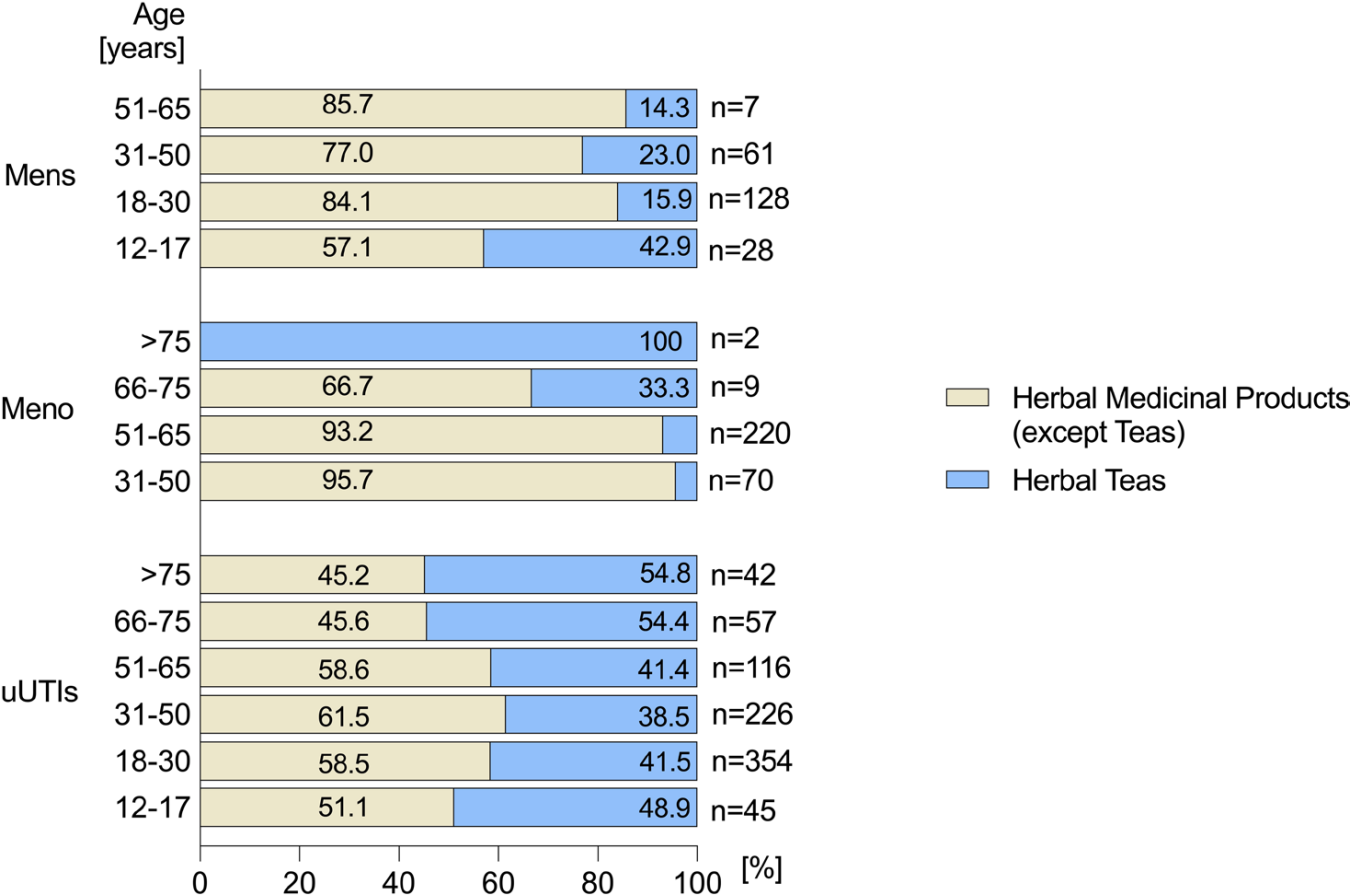
Preferences for herbal medicinal products except Teas (HMPs‐eT) or herbal teas (HTs) in relation to age and type of ailment—For menstrual complaints (Mens, *n* = 222), the preference for HTs decreases with increasing age, whereas the opposite was the case for menopausal symptoms (Meno, *n* = 301). Women with uncomplicated urinary tract infections (uUTIs, *n* = 840) utilised both pharmaceutical forms likewise. Across all three indications, HMPs‐eT are generally preferred.

Among teenagers (12–17 years) with menstrual complaints (Mens), the use of HMPs‐eT and HTs is nearly balanced (57.1% vs. 42.9%), with a slight preference for HMPs‐eT. In contrast, more than three quarters of adult women preferred (77%–86%) HMPs‐eT (Figure 4). Additionally, teenagers (12–17 years) applied statistically significantly more often HTs than women aged 18–30 years (*p* < 0.01**, *r* = 0.256), as evidenced by Mann–Whitney‐*U*‐test (Table S1).

The vast majority of women with menopausal complaints in the age groups 31–50 years (95.7%) or 51–65 years (93.2%) preferred HMPs‐eT. About two‐thirds of the women over 66 years were using HMPs‐eT (66.7%, *n* = 6), while the remaining one third preferred HTs. Women aged 31–65 years with menopausal symptoms showed a statistically significant preference for HMPs‐eT compared to women aged 66–75 years (*p* < 0.01**, *r* = 0.346) and those over 75 years (*p* < 0.01**, *r* = 0.615), as evidenced by the Mann–Whitney *U* test.

Regarding uncomplicated uUTIs, the use of HMPs‐eT and HTs was quite balanced throughout all age groups (Figure 4). This applies to the age groups of teenagers (51.1% vs. 48.1%) and women over 66 years (about 45.5% vs. 55.5%). Nonetheless the general trend to apply HTs mentioned already in the chapter before (‘Preferences for certain pharmaceutical forms’) starting at the age of 66 is visible here.

### 3.5 The Effect of Severity of Symptoms

**FIGURE 5:**
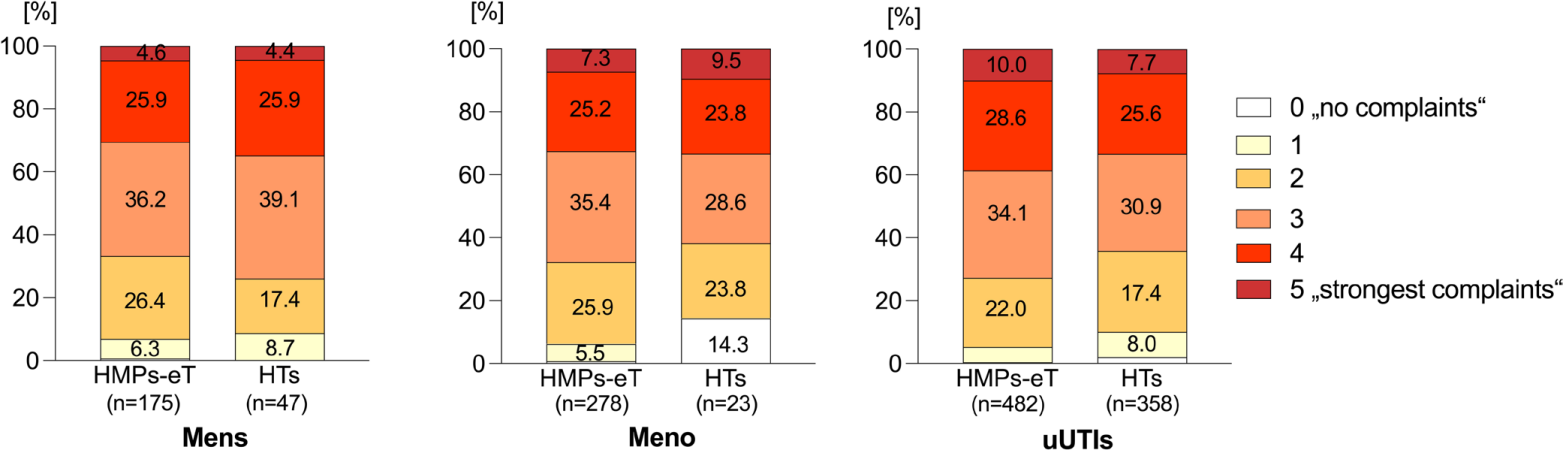
Treatment with herbal medicinal products except Teas (HMPs‐eT) and herbal teas (HTs) in relation to severity of symptoms—no preferences for a certain pharmaceutical form could be observed; menstrual (Mens, *n* = 222) and menopausal complaints (Meno; *n* = 301) and uncomplicated urinary tract infections (uUTIs, *n* = 840).

We did not observe any trend regarding the preference for a certain pharmaceutical form in the context of symptom severity. This applies for all types of ailments (Figure 5, Table S2).

### 3.6 Frequency of Use of Medication in Relation to Pharmaceutical Form

The majority of women with *menstrual and menopausal complaints* applied HMPs‐eT ‘daily’ (86.5%, *n* = 148; 97.1%, *n* = 267 respectively), and only a small portion (13.5%, *n* = 23; 2.9%, *n* = 8) ‘if required’.

Among women who preferred HTs for the alleviation of menstrual symptoms (*n* = 47), around two thirds (66.7%, *n* = 32) took it daily, whereas one third (33.3%, *n* = 15) used HTs ‘if required’.

**FIGURE 6:**
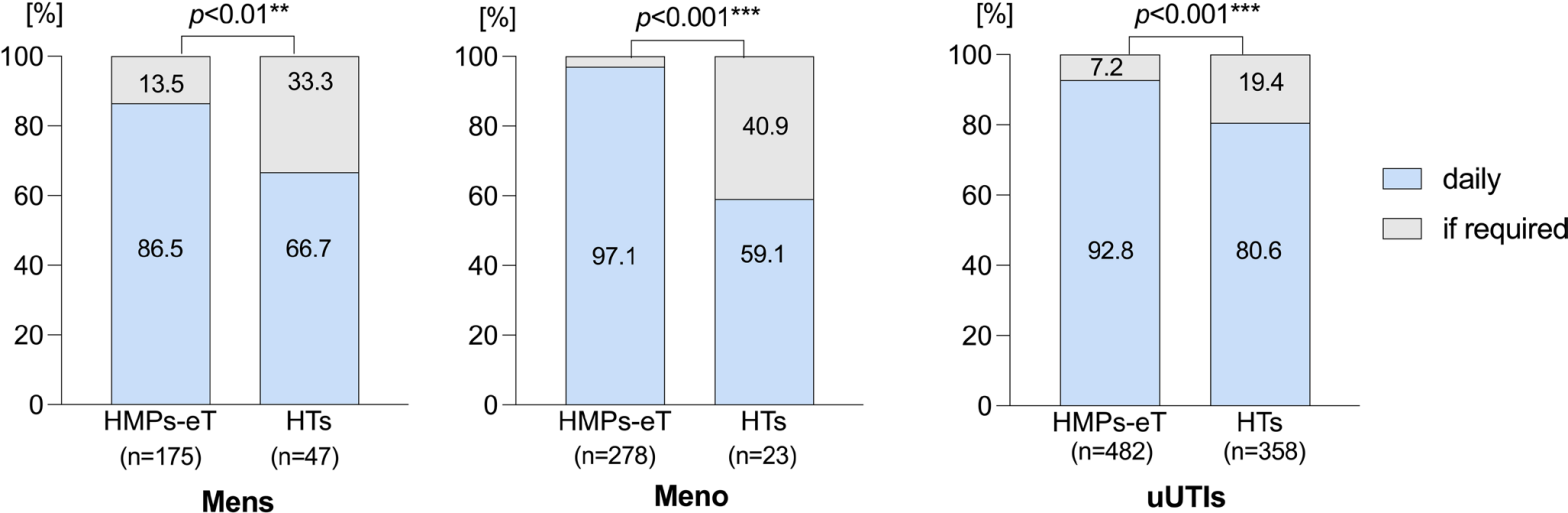
Treatment habits of herbal medicinal products except Teas (HMPs‐eT) and herbal teas (HTs) for menstrual (Mens, *n* = 222) and menopausal (Meno, *n* = 301) complaints and uncomplicated urinary tract infections (uUTIs, *n* = 840)— There was a trend to apply HMPs‐eT ‘daily’ and HTs ‘if required’, irrespective of the type of ailment.

Regarding the small share of women who treated their menopausal symptoms with HTs (*n* = 23), only slight differences in treatment habits could be observed, with about 60% using HTs ‘daily’ and about 40% ‘if required’ (Figure 6).

There was a visible trend regarding the medical intervention against *uUTIs*: HMPs were applied mostly ‘daily’ regardless of the pharmaceutical form, with more than 90% who took HMPs‐eT (92.8%, *n* = 439) and more than 80% who used HTs (80.6%, *n* = 282).

Six women with menstrual complaints, four women with menopausal complaints, and 17 women with uUTIs did not give any information about the use of HMPs‐eT or HTs.

Irrespective of the type of complaint, women preferred to apply HMPs‐eT rather ‘daily’, and HTs rather ‘if required’, however, to different extents. The differences were statistically significant, as evidenced by the chi‐squared test whereby the effect size for uUTIs was quite small (Mens: *p* < 0.01**, *V* = 0.212, Meno: *p* < 0.001***, *V* = 0.428; uUTIs: *p* < 0.001**, *V* = 0.184) (Figure 6, Table S3).

### 3.7 Long‐Term Versus Short‐Term Use of Medication in Relation to Pharmaceutical Form

**FIGURE 7:**
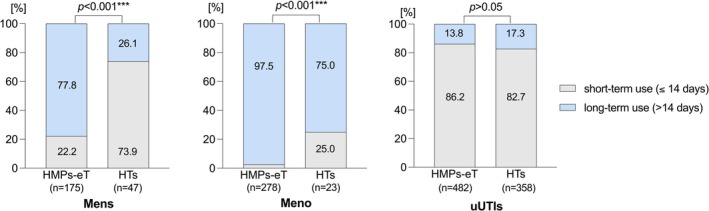
Duration of treatment with herbal medicinal products except Teas (HMPs‐eT) and herbal teas (HTs) for menstrual (Mens, *n* = 222) and menopausal complaints (Meno, *n* = 301) and uncomplicated urinary tract infections (uUTIs, *n* = 840)—For Mens and Meno, HMPs‐eT were predominantly used for a longer period of time, whereas HTs were more often applied for a shorter time period. In contrast, for uUTIs, both HMPs‐eT and HTs were mainly used for short periods.

In our sample, a general trend to apply HMPs‐eT for a longer period was visible for menstrual and menopausal complaints: In detail, about three quarters (77.8%, *n* = 133) of women with menstrual complaints and almost all (97.5%, *n* = 38) women with menopausal symptoms applied HMPs‐eT for a longer period (Figure 7).

In contrast, about three quarters of the women with *menstrual complaints* (73.9%, *n* = 34) used HTs for a shorter time period, while the trend was more towards a long‐term use by women with *menopausal symptoms* (75.0%, *n* = 18). The differences were statistically significant for menstrual (*p* < 0.001***, *V* = 0.449) and menopausal (*p* < 0.001***, *V* = 0.286) complaints, with considerable effect sizes (Figure 7 and Table S4).

Women with *uUTIs* applied HMPs predominantly for short‐time periods (86.2%, *n* = 401; 82.7%, *n* = 283) regardless of the pharmaceutical form (HMPs‐eT or HTs) without any statistically significant differences.

In total, five women with menstrual complaints, six women with menopausal complaints, and 33 women with uUTIs did not give any information about the long‐term versus short‐term use.

### 3.8 Preferences Regarding Specific Plant Drug for Certain Gynaecological Conditions

Most women with *menstrual complaints* (97.1%; *n* = 170) using mono‐preparations of HMPs‐eT preferred *Vitex agnus‐castus* L. to treat their symptoms. *Actaea racemosa* L. was used only by 1.7% (*n* = 3), and both *Alpinia officinarum* Hance and *Arctostaphylos uva‐ursi* (L.) Spreng. were used by only one participant, respectively (0.6%; *n* = 1).

Women applying HTs chose a big variety of medicinal plants which differed from those contained in HMPs‐eT. Among the mono‐preparations, *Argentina anserina* (L.) Rydb. (23.4%; *n* = 11), *Alchemilla vulgaris* L. (19.1%; *n* = 9), *Matricaria chamomilla* L. (12.8%; *n* = 6) and *Foeniculum* Mill. (10.6%; *n* = 5) were the most prevalent (Table 2). Less frequently, mono‐preparations containing *Mentha × piperita* L. (6.4%; *n* = 3), *Hypericum perforatum* L. (4.3%; *n* = 2), *Achillea millefolium* L. (4.3%; *n* = 2), *Equisetum arvense* L. (2.1%; *n* = 1), *Viola tricolor* L. (2.1%; *n* = 1) and *Cinnamomum verum* J. Presl (2.1%; *n* = 1) were used.

HTs with combinations were less common for the treatment of menstrual complaints, and if the following three specific combinations were used: a combination of *Betula pendula* Roth, *Arctostaphylos uva‐ursi* (L.) Spreng. and *Achillea millefolium* L. (6.4%; *n* = 3), a mixture of *Foeniculum vulgare* Mill., *Carum carvi* L. and *Pimpinella anisum* L. (4.3%; *n* = 2), and a product combining *Vitex agnus‐castus* L. and *Alchemilla vulgaris* L. (2.1%; *n* = 1). An overview is presented in Table 1.

**TABLE 1:** Overview of medicinal plants utilised for the treatment of menstrual complaints (Mens), categorised according to herbal medicinal products (except Teas, HMPs‐eT) and herbal teas (HTs), and further grouped as mono‐preparations^a^ or combi‐preparations^b^.

	Medicinal plants	HMPs‐eT (*n* = 175)	HTs (*n* = 47)
Mono‐preparations^a^	*Vitex agnus‐castus* L.	97.1% (*n* = 170)	—
	*Actaea racemosa* L.	1.7% (*n* = 3)	—
	*Alpinia officinarum* Hance	0.6% (*n* = 1)	—
	*Arctostaphylos uva‐ursi* (L.) Spreng.	0.6% (*n* = 1)	—
	*Argentina anserina* (L.) Rydb.	—	23.4% (*n* = 11)
	*Alchemilla vulgaris* L.	—	19.1% (*n* = 9)
	*Matricaria chamomilla* L.	—	12.8% (*n* = 6)
	*Foeniculum vulgare* Mill.	—	10.6% (*n* = 5)
	*Mentha × piperita* L.	—	6.4% (*n* = 3)
	*Hypericum perforatum* L.	—	4.3% (*n* = 2)
	*Achillea millefolium* L.	—	4.3% (*n* = 2)
	*Equisetum arvense* L.		2.1% (*n* = 1)
	*Viola tricolor* L.	—	2.1% (*n* = 1)
	*Cinnamomum verum* J. Presl	—	2.1% (*n* = 1)
Combi‐preparations^b^	*Betula pendula* Roth, *Arctostaphylos uva‐ursi* (L.) Spreng., *Achillea millefolium* L.	—	6.40% (*n* = 3)
	*Foeniculum vulgare* Mill., *Carum carvi* L., *Pimpinella anisum* L.	—	4.30% (*n* = 2)
	*Vitex agnus‐castus* L., *Alchemilla vulgaris* L.	—	2.10% (*n* = 1)

^a^ *Mono‐preparation*: containing only one medicinal plant extract.

^b^ *Combi‐preparation*: containing extracts from more than one medicinal plant.

Women treating their *menopausal complaints* with HMPs‐eT chose the following seven different herbal drugs either as mono‐preparations or in combinations: Most frequently *Actaea racemosa* L. (79.1%; *n* = 220), followed by *Vitex agnus‐castus* L. (9.7%; *n* = 27) and less common but still worth mentioning *Rheum rhaponticum* L. (2.8%; *n* = 8), *Hypericum perforatum* L. (1.1%; *n* = 3) and *Lavandula angustifolia* Mill. (0.4%; *n* = 1). Among the combinations consisting of standardised extracts from the plants *Actaea racemosa* L. and *Hypericum perforatum* L. (6%; *n* = 17) was used most frequently. Only few women reported using combinations of *Hypericum perforatum* L. and *Valeriana officinalis* L. (0.7%; *n* = 2) or *Valeriana officinalis* L. and *Humulus lupulus* L. and *Passiflora incarnata* L. (0.4%; *n* = 1).

In the few cases where HTs were applied to alleviate the symptoms of menopausal complaints, the most commonly used mono‐preparations contained *Salvia officinalis* L. (26.1%; *n* = 6), *Alchemilla vulgaris* L. (21.7%; *n* = 5) and *Viscum album* L. (13.0%; *n* = 3) (Table 2). *Achillea millefolium* L. (8.7%; *n* = 2), *Argentina anserina* (L.) Rydb. (4.3%; *n* = 1), *Solidago virgaurea* L. (4.3%; *n* = 1), *Trifolium pratense* L. (4.3%; *n* = 1), *Vitex agnus‐castus* L. (4.3%; *n* = 1) or *Crataegus monogyna* Jacq. (4.3%; *n* = 1) were also used sporadically. Among the few cases in which combination HTs were used, the most common preparations were *Vitex agnus‐castus* L. and *Alchemilla vulgaris* L. (4.3%; *n* = 1) and *Betula pendula* Roth and *Arctostaphylos uva‐ursi* (L.) Spreng. and *Achillea millefolium* L. (4.3%; *n* = 1). An overview is presented in Table 2.

**TABLE 2:** Overview of medicinal plants utilised for the treatment of menopausal complaints (Meno), categorised according to herbal medicinal products except Teas (HMPs‐eT) and herbal teas (HTs), and further grouped as mono‐preparations^a^ or combi‐preparations^b^.

	Medicinal plants	HMPs‐eT *n* = 278	HTs (*n* = 23)
Mono‐preparations^a^	*Actaea racemosa* L.	79.10% (*n* = 220)	—
	*Vitex agnus‐castus* L.	9.70% (*n* = 27)	4.30% (*n* = 1)
	*Rheum rhaponticum* L.	2.90% (*n* = 8)	—
	*Hypericum perforatum* L.	0.70% (*n* = 2)	—
	*Lavandula angustifolia* Mill.	0.40% (*n* = 1)	—
	*Salvia officinalis* L.	—	26.10% (*n* = 6)
	*Alchemilla vulgaris* L.	—	21.70% (*n* = 5)
	*Viscum album* L.	—	13.00% (*n* = 3)
	*Achillea millefolium* L.	—	8.70% (*n* = 2)
	*Argentina anserina* (L.) Rydb.	—	4.30% (*n* = 1)
	*Solidago virgaurea* L.	—	4.30% (*n* = 1)
	*Trifolium pratense* L.	—	4.30% (*n* = 1)
	*Crataegus monogyna* Jacq.	—	4.30% (*n* = 1)
Combi‐preparations^b^	*Actaea racemosa* L., *Hypericum perforatum* L.	6.10% (*n* = 17)	—
	*Hypericum perforatum* L., *Valeriana officinalis* L.	0.70% (*n* = 2)	—
	*Valeriana officinalis* L., *Humulus lupulus* L., *Passiflora incarnata* L.	0.40% (*n* = 1)	—
	*Vitex agnus‐castus* L., *Alchemilla vulgaris* L.	—	4.30% (*n* = 1)
	*Betula pendula* Roth, *Arctostaphylos uva‐ursi* (L.) Spreng., *Achillea millefolium* L.	—	4.30% (*n* = 1)

^a^ *Mono‐preparation*: containing only one medicinal plant extract.

^b^ *Combi‐preparation*: containing extracts from more than one medicinal plant.

Among the women who applied HMPs‐eT to treat their symptoms of uUTIs, the majority used a mono‐preparation of *Arctostaphylos uva‐ursi* (L.) Spreng. (44.2%; *n* = 213). HMPs‐eT containing only *Solidago virgaurea* L. were used less frequently (8.7%; *n* = 42). Other mono‐preparations were also used; however, their frequency was below 1% in our data sample.

A quarter of the patients used HMPs‐eT containing a combination of *Centaurium erythraea* Rafn and *Levisticum officinale* W.D.J. Koch and *Salvia rosmarinus* Spenn. (25.5%; *n* = 123) and a minor share used a mixture of *Tropaeolum majus* L. and *Armoracia rusticana* G. Gaertn., B. Mey. and Scherb. (11.2%; *n* = 54) or a combination of *Betula pendula* Roth and *Arctostaphylos uva‐ursi* (L.) Spreng. and *Achillea millefolium* L. (6.8%; *n* = 33). The frequency of further combinations was below 1%.

Regarding the use of HTs, the majority of women decided to take a combination of *Betula pendula* Roth and *Arctostaphylos uva‐ursi* (L.) Spreng. and *Achillea millefolium* L. (83.3%; *n* = 299). A minor share applied mono‐preparations of HTs containing only *Arctostaphylos uva‐ursi* (L.) Spreng. (5%; *n* = 18), or *Urtica urens* L. (6.1%; *n* = 22) or *Betula pendula* Roth (1.4%; *n* = 5). An overview is presented in Table 3.

**TABLE 3:** Overview of medicinal plants utilised for the treatment of uncomplicated urinary tract infections (uUTIs), categorised according to herbal medicinal products (except Teas, HMPs‐eT) and herbal teas (HTs), and further grouped as mono‐preparations^a^ or combi‐preparations^b^.

	Medicinal plants	HMPs‐eT (*n* = 482)	HTs (*n* = 359)
Mono‐preparations^a^	*Arctostaphylos uva‐ursi* (L.) Spreng.	44.20% (*n* = 213)	5.00% (*n* = 18)
	*Solidago virgaurea* L.	8.70% (*n* = 42)	0.80% (*n* = 3)
	*Urtica urens* L.	—	6.10% (*n* = 22)
	*Betula pendula* Roth	—	1.40% (*n* = 5)
	*Cucurbita pepo* L.	0.80% (*n* = 4)	—
	*Echinacea purpurea* (L.) Moench	0.60% (*n* = 3)	—
	*Equisetum arvense* L.	—	0.80% (*n* = 3)
	*Matricaria chamomilla* L.	0.40% (*n* = 2)	0.80% (*n* = 3)
	*Orthosiphon aristatus* (Blume) Miq.	0.20% (*n* = 1)	—
	*Rosa canina* L.	—	0.30% (*n* = 1)
	*Ononis spinosa* L.	—	0.30% (*n* = 1)
	*Vaccinium myrtillus* L.	—	0.30% (*n* = 1)
	*Senna alexandrina* Mill.	—	0.30% (*n* = 1)
	*Salvia officinalis* L.	—	0.30% (*n* = 1)
	*Epilobium angustifolium* L.	—	0.30% (*n* = 1)
Combi‐preparations^b^	*Centaurium erythraea* Rafn, *Levisticum officinale* W.D.J. Koch, *Salvia rosmarinus* Spenn.	25.5% (*n* = 123)	—
	*Tropaeolum majus* L., *Armoracia rusticana* G. Gaertn., B. Mey. and Scherb.	11.20% (*n* = 54)	—
	*Betula pendula* Roth, *Arctostaphylos uva‐ursi* (L.) Spreng., *Achillea millefolium* L.	6.80% (*n* = 33)	83.3% (*n* = 299)
	*Rhus coriaria* L., *Cucurbita pepo* L., *Humulus lupulus* L.	0.80% (*n* = 4)	—
	*Argentina anserina* (L.) Rydb., *Solidago virgaurea* L., *Equisetum arvense* L.	0.60% (*n* = 3)	—

^a^ *Mono‐preparation*: containing only one medicinal plant extract.

^b^ *Combi‐preparation*: containing extracts from more than one medicinal plant.

### 3.9 Perceived Therapeutic Effectiveness of HMPs Regardless of Plant Drug

**FIGURE 8:**
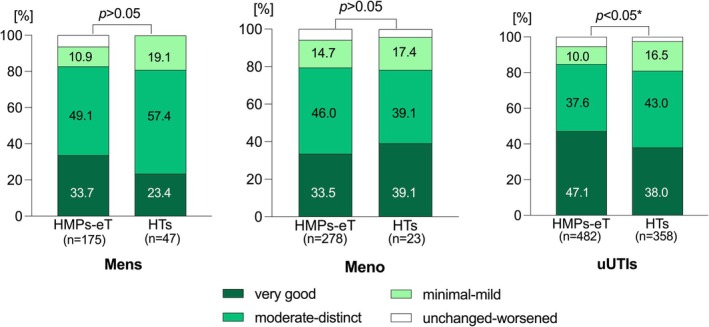
Perceived effectiveness of herbal medicinal products except Teas (HMPs‐eT) and herbal teas (HTs) for menstrual (Mens, *n* = 222) and menopausal complaints (Meno, *n* = 301) and uncomplicated urinary tract infections (uUTIs, *n* = 840) – The vast majority of women perceived an exceptional effectiveness, irrespective of type of ailment and with no substantial differences between pharmaceutical form.

Generally, women with gynaecological complaints perceived the therapeutic benefit of HMPs as very good, regardless of type of ailment and pharmaceutical form (Figure 8).

In detail, the vast majority of women with menstrual complaints reported remarkable benefits of HMPs‐eT between ‘moderate to distinct’ (49.1%; *n* = 86) and ‘very good’ (33.7%; *n* = 59), and only less than 10% (6.3%, *n* = 11) did not perceive any therapeutic effectiveness. A similar picture could be observed with HTs (‘moderate–distinct’ 57.4%; *n* = 27; ‘very good’ 23.4%, *n* = 11). There were no statistically significant differences regarding the perceived effectiveness between HMPs‐eT and HTs (Mann–Whitney *U* test: *p* > 0.05).

Concerning menopausal complaints, around 80% of women rated the effectiveness of both pharmaceutical forms (HMPs‐eT and HTs) as ‘very good’ (33.5%, *n* = 93; 39.1%, *n* = 9 respectively) or ‘moderate‐distinct’ (46.0%, *n* = 128; *n* = 39.1%, *n* = 9). About 15% perceived at least ‘minimal to mild’ improvements (14.7%; *n* = 41, 17.4%, *n* = 4 respectively) and less than 6% stated no relief of their symptoms (5.8%; *n* = 16; 4.3%, *n* = 1 respectively). Again, no statistically significant differences regarding the perceived effectiveness between HMPs‐eT and HTs could be observed (Mann–Whitney *U* test: *p* > 0.05) (Figure 8).

Concerning the treatment of uUTIs, there were statistically significant differences in the perception of the therapeutic effectiveness between HMPs‐eT and HTs (Mann–Whitney *U* test: *p* < 0.05*), however with a very small effect size (*r* = 0.0834) (Figure 8 and Table S5).

### 3.10 Perceived Therapeutic Effectiveness of Specific Plant Drugs

#### 3.10.1 Treatment of Menstrual Complaints, With *Vitex agnus‐castus* L. (VAC)

We analysed only the use of VAC in the pharmaceutical form of a HMPs‐eT (*n* = 170) because the number of cases regarding all other plant drugs applied for the treatment of menstrual complaints was too small (Table 1).

Patients with menstrual problems treating their symptoms with HMPs‐eT containing VAC perceived the effectiveness mostly as ‘very good’ (34.1%, *n* = 58) or moderate‐distinct (48.8%, *n* = 83). Only a few reported on a ‘minimal‐mild’ effectiveness (10.6%, *n* = 18) or that their symptoms were unchanged (6.4%; *n* = 11).

#### 3.10.2 Treatment of Menopausal Symptoms With *Actaea racemosa* L. (CR), *Vitex agnus‐castus* L. (VAC) and *Hypericum perforatum* L. (HP)

We analysed only the use of HMPs‐eT containing mono‐preparations of CR, VAC or a combination of CR & HP, because the number of cases regarding all other plant drugs or HTs applied for the treatment of menopausal complaints was too small (Table 2).

The vast majority of patients in the remaining sample reported that the therapeutic benefit of CR was notable with ‘very good’ (33.6%; *n* = 74) or ‘moderate‐distinct’ (46.8%; *n* = 103). A minimal to mild effectiveness was perceived by a minor share (14.1%, *n* = 31) and only a few (5.5%, *n* = 12) did not experience any changes in symptoms.

Similar results could be observed with the use of VAC: Around one third of the women rated the improvement in symptoms as very good (25.9%, *n* = 7), and about half of them as ‘moderate to distinct’ (48.1%, *n* = 13). Again, a minor share reported a slight (14.8%; *n* = 4) or no noticeable improvement of their symptoms (11.1%; *n* = 3).

Among the women applying the combination of CR and HP, all women without any exception reported on therapeutic benefits, with the majority considering the effectiveness to be ‘very good’ (41.2%, *n* = 7), or ‘moderate‐distinct’ (35.3%, *n* = 6). The remaining share rated the effectiveness at least as ‘minimal‐mild’ (23.5%, *n* = 4).

#### 3.10.3 Treatment of uUTIs With a Variety of Plant Drugs

We analysed only subgroups with a sample size *n* > 15 (Table 3) and all other groups or single cases were considered to be too small and therefore excluded from our analysis.

Women with uUTIs who used *Arctostaphylos uva‐ursi* (L.) Spreng. as HMPs‐eT or HTs reported considerable therapeutic benefits, regardless of the pharmaceutical form. Nearly half of those preferring HMPs‐eT (46.7%; *n* = 100), but only a third of those applying HTs (27.8%, *n* = 5), rated the effectiveness as ‘very good’. In contrast, ‘moderate‐distinct’ effectiveness was reported by half of the women (50%, *n* = 9) preferring HTs and by about one third (38.8%, *n* = 82) of those preferring HMPs‐eT. Regarding those fewer patients who experienced only a ‘minimal‐mild’ improvement in symptoms, the proportion of those using HTs was twice as large (HMPs‐eT: 9.9%, *n* = 21 versus HT: 22.2%, *n* = 4) as those using HMPs‐eT. Only a small share of women using HMPs‐eT reported no effectiveness (4.7%; *n* = 10).

At first glance, it appears that users of HMPs‐eT perceive a greater therapeutic benefit than those who use HTs, but this trend is statistically not significant (*p* = 0.137; *r* = 0.098, Mann–Whitney *U* test) (Table S6).

In the group of patients who preferred *Solidago virgaurea* L.‐based preparations the vast majority perceived the effectiveness as ‘very good’ (61.9%; *n* = 26) or at least as ‘moderate‐distinct’ (31%; *n* = 13). Reports of ‘minimal‐mild’ improvements (2.4%; *n* = 1) or no changes in symptoms (4.8%; *n* = 2) were rare.

Regarding the application of *Urtica urens* L. all women without any exception reported on therapeutic benefits with the majority considering the effectiveness to be ‘very good’ (28.6%; *n* = 6) or at least ‘moderate‐distinct’ (47.6%, *n* = 10). The remaining quarter (23.8%; *n* = 5) described the effect of the drug intervention as ‘minimal‐mild’.

The utilisation of all three mono‐preparations generated similar satisfaction among users regarding therapeutic effectiveness and none of the preparations are superior to the other.

The three combinations containing *Centaurium erythraea* Rafn and *Levisticum officinale* W.D.J. Koch and *Salvia rosmarinus* Spenn (CLS) or *Tropaeolum majus* L. and *Armoracia rusticana* G. Gaertn., B. Mey. and Scherb. (TC) or *Betula pendula* Roth and *Arctostaphylos uva‐ursi* (L.) Spreng. and *Achillea millefolium* L. (UVA) used for the treatment of uUTIs were rated as quite effective by the women in our sample, with no significant differences.

The majority of women rated all combi‐treatments with ‘very good’ (37%–48.5% of the cases) to ‘moderate‐distinct’ (24.2%–42.6%) effectiveness. Only a few women did not perceive any symptom relief upon application of the HMPs (2.7%–9.1%).

The combination of *Betula pendula* Roth and *Arctostaphylos uva‐ursi* (L.) Spreng. and *Achillea millefolium* L. (UVA) was used in both pharmaceutical forms; however, the women did not report any significant differences regarding the effectiveness (*p* > 0.05; *r* = 0.01, Mann–Whitney *U* test) (Table S5). Again, none of the preparations is superior to the other, as evidenced by statistical analysis.

### 3.11 Perception of Undesired Drug Events

The vast majority of women (more than 90%) did not report the occurrence of undesirable drug events caused by HMPs and 7% perceived tolerability issues that did not impair them. Adverse events that caused ‘significant impairment’ or whose ‘severity outweigh the perceived effectiveness’ were reported by only a few participants (1.5% or 0.4% respectively) (Table 4). None of the perceived undesired events reported by the patients have been life‐threatening.

**TABLE 4:** Overview of undesired tolerability issues**—**adverse events were reported rarely (study sample: *N* = 1363).

	None	No impairment	Significant impairment	Outweigh effectiveness
*n* = 1363	90.8% (*n* = 1238)	7.3% (*n* = 99)	1.5% (*n* = 20)	0.4% (*n* = 6)

Regarding the safety and tolerability of HMPs, significant tolerability issues were reported mostly for *Actaea racemosa* L. Mild side effects without any impairment have been observed for all applied HMPs; however, more frequently for *Actaea racemosa* L., *Arctostaphylos uva‐ursi* (L.) Spreng., and *Urtica urens* L., however the first three drugs mentioned also occurred most frequently in our data (Table 5).

**TABLE 5:** Frequencies of undesired tolerability issues of selected herbal medicinal product‐preparations grouped in mono‐preparations (Mono) and combi‐preparations (Combi)—The few reported undesired events were mild and non‐impairing. Significant tolerability issues were rare and mainly associated with CR.

			No impairment	Significant impairment	Outweigh effectiveness
VAC	Mono	*n* = 198	11.6% (*n* = 23)	0% (*n* = 0)	0.5% (*n* = 1)
	Combi	*n* = 0	—	—	—
CR	Mono	*n* = 223	8.1% (*n* = 18)	5.4% (*n* = 12)	0.9% (*n* = 2)
	Combi	*n* = 17	0.0% (*n* = 0)	5.8% (*n* = 1)	0.0% (*n* = 0)
UVA	Mono	*n* = 232	6.9% (*n* = 16)	0.9% (*n* = 2)	0.4% (*n* = 1)
	Combi	*n* = 336	5.7% (*n* = 19)	0.9% (*n* = 3)	0.0% (*n* = 0)
U	Mono	*n* = 21	9.5% (*n* = 2)	0.0% (*n* = 0)	0.0% (*n* = 0)
	Combi	*n* = 0	—	—	—
SOL	Mono	*n* = 46	4.3% (*n* = 2)	2.2% (*n* = 1)	2.2% (*n* = 1)
	Combi	*n* = 0	—	—	—
CLS	Mono	*n* = 0	—	—	—
	Combi	*n* = 123	4.9% (*n* = 6)	0.8% (*n* = 1)	0.0% (*n* = 0)
TC	Mono	*n* = 0	—	—	—
	Combi	*n* = 54	11.1% (*n* = 6)	0.0% (*n* = 0)	1.9% (*n* = 1)

Abbreviations: VAC = *Vitex agnus‐castus* L; CR = *Actaea racemosa* L; CR in combination with *Hypericum perforatum* L; UVA = *Arctostaphylos uva‐ursi* (L.) Spreng; UVA in combination with *Betula pendula* Roth and *Achillea millefolium* L; SOL = *Solidago virgaurea* L; CLS = *Centaurium erythraea* Raf; *Levisticum officinale* W.D.J. Koch*; Salvia rosmarinus* Spenn; TC = *Tropaeolum majus* L.; *Armoracia rusticana* G. Gaertn., B. Mey.; Scherb.

## 4 Discussion

Data from the real world of medical care in everyday practice are an extremely valuable addition to results from clinical studies, which are obtained under the ideal conditions of a clinical trial with a carefully selected patient population and often cannot be transferred completely to the reality of medical healthcare. In addition, real‐world data, especially PROs, play an important role in the context of symptoms that cannot be measured by instruments or laboratory diagnostics.

In our study, we analysed a sample consisting of PROs (*n* = 1363) from the pharmaco‐epidemiological database PhytoVIS and provide valuable insights into the everyday HMP utilisation for the treatment of gynaecological complaints. In addition, we delineate so far unknown factors that influence HMP utilisation.

It is generally known that HMPs can be used in self‐medication for mild to moderate, non‐life‐threatening symptoms (Wegener et al. 2021). HMPs are considered a gentle, well‐tolerated alternative to non‐herbal medicines, especially for conditions that do not require aggressive pharmacological intervention (Salm et al. 2023; Cerqueira et al. 2017). We therefore assumed that the women in our sample would also predominantly classify their symptoms as mild to moderate (severity = 1–3). Interestingly, regardless of the type of ailment, about a quarter of the patients in our dataset reported having severe to very severe symptoms, which they nevertheless treated with HMPs. The utilisation of HMPs for very severe symptoms indicates that patients have a high level of confidence regarding the effectiveness, even though the level of distress is high. This underlines the importance of HMPs as an alternative treatment option in everyday practice.

Regarding the pharmaceutical form, we observed that regardless of age, women with symptoms of uUTIs showed a higher tendency to use HTs in respect to the other two types of ailments. Likewise, young teenagers with menstrual complaints (12–17 years) use HTs significantly more often than women between 18 and 30 years. This could be due to the fact that the parents of teenagers have an influence on the treatment of their underaged daughters' menstrual symptoms and make sure that the drug therapy is particularly gentle and effective, such as treatment with HMPs (Schellenberg 2001; He et al. 2009; Momoeda et al. 2014). Advice from pharmacists certainly plays a significant role here—especially with regard to our PhytoVIS data, which was collected through a survey of customers and patients in pharmacies (Wegener et al. 2021). Additionally, women from the age of 66 years onwards with menopausal symptoms also preferred HTs. One reason for this could be that older patients have to take several medications at the same time (polypharmacy) (Lappe et al. 2022), which could negatively influence the willingness to take (additional) medications. In this context, the preference for HTs appears to be a possible strategy for patients to avoid or minimise potential drug interactions, as HTs are considered harmless in the population. A further reason could be that most women over the age of 66 are in postmenopause. These women possibly consider taking medication for perimenopausal symptoms to be unnecessary and prefer to drink a HT.

Regarding the treatment of menstrual and menopausal complaints, we observed less variability with respect to the medicinal plants when using HMPs‐eT compared with HTs.

Most women preferred *Vitex‐agnus castus* L. for menstrual complaints and *Actaea racemosa* L. for menopausal complaints. This could primarily be due to their proven efficacy in clinical trials, where *Vitex‐agnus castus* L. (Schellenberg 2001; He et al. 2009) and *Actaea racemosa* L. (Mohammad‐Alizadeh‐Charandabi et al. 2013; Osmers et al. 2005) had significantly better therapeutic effects compared to placebo or non‐herbal medicines. An important role in the decision for ‘the right’ HMP could be that patients trust the sound advice they receive in pharmacies (Welz et al. 2019a). Although only a few women used HTs (Mens *n* = 47; Meno *n* = 23), the variety of medicinal plants is large. *Argentina anserina* (L.) Rydb. dominated as the most frequently used medicinal plant for menstrual complaints, while *Salvia officinalis* L. was used most frequently for menopausal complaints. Compared to *Vitex‐agnus castus* L. and *Actaea racemosa* L., where the application is specifically limited to either menstrual or menopausal symptoms respectively, *Argentina anserina* (L.) Rydb. and *Salvia officinalis* L. are used for a variety of symptoms that go beyond the treatment of certain gynaecological symptoms. Nevertheless, *Argentina anserina* (L.) Rydb. is used for ‘mild dysmenorrhea complaints’ (BGA/BfArM KEM 1985) or *Salvia officinalis* L. for ‘excessive sweating’ (HMPC 2016). In contrast, we observed a wide range of medicinal plants in the treatment of uUTIs across both pharmaceutical forms. While the combination of *Betula pendula* Roth, *Arctostaphylos uva‐ursi* (L.) Spreng. and *Achillea millefolium* L. dominates among the HTs, there are four herbal mono‐ or combi‐preparations used among the HMPs‐eT (see Table 3).

Our analysis of treatment habits revealed that regardless of the type of gynaecological complaint, all HMPs were predominantly taken daily. Although HTs tended to be used more frequently as needed, the frequency of use varied depending on the type of ailment. This could be because HMPs‐eT are mainly available in pharmacies, where advice is given by specialist staff at the time of dispensing and regular use is specifically advised. In comparison, HTs can also be purchased in drugstores or supermarkets, where no advice is usually given, which could possibly lead to more flexible and less regular use.

The differences we observed in the duration of use of HMPs (short‐term use vs. long‐term use) can be interpreted in the light of recommendations in the guidelines and the extent of symptoms: For menstrual complaints, the longer duration of intake of HMPs‐eT is in line with the recommendations in the HMPC monographs (Wenigmann 2017) in order to achieve a therapeutic effect. The shorter intake of HTs is in line with the persistence of the symptoms which is usually between two to six days (Schoep et al. 2019; Kural et al. 2015).

During menopause, the symptoms are no longer cycle‐dependent but result from permanent hormonal fluctuations that can be continuously noticeable for patients. This could be the reason why women predominantly use both HMPs‐eT and HTs for longer than 14 days. In the treatment of uUTIs, both pharmaceutical forms are used for a shorter period without any difference, which is due to the fact that the symptoms of acute cystitis are limited to one (maximum 2) week(s).

The women in our sample perceived the therapeutic effectiveness of HMPs as very good regardless of the plant drug, the type of ailment and the pharmaceutical form. For the treatment of menopausal complaints, the combination of *Actaea racemosa* L. and *Hypericum perforatum* L. was perceived superior to monopreparations of *Actaea racemosa* L. or *Vitex‐agnus castus* L. Women with menstrual complaints reported a significant symptom relief upon treatment with *Vitex‐agnus castus* L. and finally, for the treatment of uUTIs, monopreparations of *Urtica urens* L. and a combi‐preparation of *Centaurium erythraea* Rafn and *Levisticum officinale* W.D.J. Koch and *Salvia rosmarinus* Spenn were perceived as best regarding therapeutic benefits.

Besides the excellent therapeutic effectiveness of HMPs, undesired drug effects were reported in only about 10% of the cases and most of them were so minor that they did not affect everyday life. A minority of 2% of the women in our database (26 out of 1363) experienced significant impairments, temporally related to the intake, although the cause is not known.

Regarding the treatment of uUTIs as an infectious disease, the increasing problem of antibiotic resistance intensifies the need for research into effective treatment alternatives. The updated S3 guideline from 2024 recommends careful consideration of antibiotic therapy to avoid unnecessary treatment and the development of resistance. Medicinal plants such as *Arctostaphylos uva‐ursi* (L.) Spreng. and the combination of *Centaurium erythraea* Rafn, *Levisticum officinale* W.D.J. Koch and *Salvia rosmarinus* Spenn. are mentioned as alternatives ((Hrsg.) DGfUeV 2024). These recommendations underline the relevance of alternative therapeutic approaches and stimulate research into herbal agents as potential substitutes for antibiotics.

When interpreting these results, it must be considered that they reflect the subjective perceptions of the patients. Since the self‐reported information was collected retrospectively, it must be acknowledged that the data may be subject to recall bias.

In addition, the sample analysed is not representative of the general population in Germany, as the surveys were mainly conducted in western Germany and mainly in pharmacies (Wegener et al. 2021). The salary differences between eastern and western Germany could have an impact on the utilisation of HMPs as these are largely not reimbursed by statutory health insurances.

Furthermore, our sample consists only of women who use HMPs and a corresponding control group consisting of women who did not use HMPs did not exist in the PhytoVIS database. The absence of control or comparator groups restricts the ability to establish causal relationships.

A possible ‘selection bias’ must be pointed out, as the data examined contains statements and perceptions of patients made by women who specifically selected or were recommended certain HMPs. The patient group was not randomised, which means that a direct comparison of the effectiveness and tolerability of different products is not possible and was not the aim of these analyses.

## 5 Conclusion

Understanding the trends and patterns in the use of HMPs provides valuable insights into patient preferences and creates potential for integrating these treatments into conventional medical practices. By identifying the most popular and efficacious plants for certain gynaecological ailments, healthcare providers can better address the growing demand for complementary treatment options. This knowledge helps in tailoring healthcare to meet patient needs and underscores the importance of research and regulation to ensure the safety and efficacy of HMPs.

## Author Contributions

B.E.B. contributed to conceptualisation of the study, supervision of the project, drafting of the manuscript, writing of the manuscript, presentation of results, approval of the final version for submission. A.D. contributed to data handling, data analysis, presentation of results, drafting and writing of the manuscript, substantial contribution to the conception, design and interpretation of the data, approval of the final version for submission. A.J.S., T.O. and O.K. contributed to substantial contribution to the conception, design and interpretation of the data, critical revision of the manuscript for important intellectual content, approval of the final version for submission. R.M. planning and design of PhytoVIS database and the questionnaire, management of the database and the data generation, data cleaning and statistics, critical revision of the manuscript for important intellectual content, and approval of the final version for submission. All authors approve the final version of the manuscript for submission.

## Funding

The authors affiliated at Goethe University, Institute of Pharmaceutical Biology are funded by Dr. Willmar‐Schwabe.

## Ethics Statement

The Ethics Commission of the University Hospital of Cologne positively evaluated the project for the purpose of data protection and ethical evaluation or statement (reference: 14‐101). The ethical standards of the institutional and/or national research committee and the 1964 Helsinki declaration and its later amendments or comparable ethical standards involving human participants were met. Inclusion criterion was the informed consent of the patient or the caregiver before the individual interview. The study protocol of PhytoVIS is registered in the European Network of Centres for Pharmacoepidemiology and Pharmacovigilance (ENCePP).

## Conflicts of Interest

The authors declare no conflicts of interest.

## Supporting information


**Table S1:** Combined view on type of ailment and age: are there significant differences in preferences for certain pharmaceutical forms (HMPs‐eT versus HTs) between specific age groups (Mann–Whitney *U* test)?

**Table S2:** Frequencies of treatment with HMPs in relation to severity of symptoms.

**Table S3:** Treatment habits of HMPs: are there significant differences in preferences for certain pharmaceutical forms (HMPs‐eT vs. HTs) and the frequency of use (‘daily’ vs. ‘if required’)? (chi‐squared‐test).

**Table S4:** Are there significant differences in preferences for certain pharmaceutical forms (HMPs‐eT vs. HTs) and the duration of use (‘short‐term use’ vs. ‘long‐term use’)? (chi‐squared‐test).

**Table S5:** Perceived therapeutic effectiveness of HMPs: are there significant differences in preferences for certain pharmaceutical forms (HMPs e.T. vs. HTs) and the perceived therapeutic effectiveness (‘very good’, ‘moderate‐distinct’, ‘minimal‐mild’, ‘unchanged‐worsend’)? (Mann–Whitney *U*‐test).

**Table S6:** Perceived therapeutic effectiveness of specific drugs: are there significant differences in preferences for certain pharmaceutical forms (HMPs e.T. vs. HTs) and the perceived therapeutic effectiveness of specific drugs (‘very good’, ‘moderate‐distinct’, ‘minimal‐mild’, ‘unchanged‐worsend’)? (Mann–Whitney *U*‐test).

## Data Availability

Data was extracted from the pharmaco‐epidemiological database PhytoVIS which is owned by the ‘Kooperation Phytopharmaka’. Requests concerning the raw data have been addressed to the ‘Kooperation Phytopharmaka’. Analysed data supporting the results can be obtained from the corresponding author on reasonable request.
