# Supplementary material for: Trends and Patterns for the Use of Herbal Medicinal Products for Gynaecological Ailments
Source: Phytother Res. 2026 Apr 6;40(6):3580–94. doi: 10.1002/ptr.70321 (PMC13254121; doi:10.1002/ptr.70321)
Supplement: Supplementary file 1 — Table S1: Combined view on type of ailment and age: are there significant differences in preferences for certain pharmaceutical forms (HMPs‐eT versus HTs) between specific age groups (Mann–Whitney U test)? [file PTR-40-3580-s006.docx]

**Supplementary Table 1: Combined View on Type of Ailment and Age:** Are there significant differences in preferences for certain pharmaceutical forms (HMPs-eT versus HTs) between specific age groups (Mann-Whitney U test)?

| **Indication** | **Group I (*m*_Rang_)** | **Group II (*m*_Rang_)** | ***p*** | ***r*** | **N** |
| --- | --- | --- | --- | --- | --- |
| **Menstrual Complaints** | 12-17 yrs (94.50) | 18-30 yrs (73.72) | 0.002* | 0.256 | 154 |
|  | 12-17 yrs (51.07) | 31-50 yrs (42.21) | 0.057 | 0.202 | 89 |
|  | 12-17 yrs (19.00) | 51-65 yrs (14.00) | 0.168 | 0.233 | 35 |
|  | 18-30 yrs (91.84) | 31-50 yrs (98.46) | 0.241 | 0.086 | 187 |
|  | 18-30 yrs (67.06) | 51-65 yrs (66.00) | 0.911 | 0.0097 | 133 |
|  | 31-50 yrs (34.80) | 51-65 yrs (31.86) | 0.603 | 0.063 | 68 |
| **Menopausal Complaints** | 31-50 yrs (142.71) | 51-65 yrs (146.39) | 0.445 | 0.045 | 290 |
|  | 31-50 yrs (38.69) | 66-75 yrs (50.17) | 0.002* | 0.346 | 79 |
|  | 31-50 yrs (35.54) | >75 yrs (70.00) | 0.005* | 0.615 | 72 |
|  | 51-65 yrs (113.81) | 66-75 yrs (144.17) | 0.004* | 0.191 | 229 |
|  | 51-65 yrs (110.57) | >75 yrs (214.00) | 8.56E-7* | 0.330 | 222 |
|  | 66-75 yrs (5.33) | >75 yrs (9.00) | 0.218 | 0.492 | 11 |
| **Uncomplicated Urinary Tract Infections** | 12-17 yrs (213.03) | 18-30 yrs (198.34) | 0.347 | 0.047 | 399 |
|  | 12-17 yrs (147.74) | 31-50 yrs (133.66) | 0.195 | 0.079 | 271 |
|  | 12-17 yrs (85.36) | 51-65 yrs (79.31) | 0.390 | 0.068 | 161 |
|  | 12-17 yrs (49.93) | 66-75 yrs (52.74) | 0.583 | 0.054 | 102 |
|  | 12-17 yrs (42.77) | >75 yrs (45.32) | 0.586 | 0.058 | 87 |
|  | 18-30 yrs (293.92) | 31-50 yrs (285.14) | 0.469 | 0.030 | 580 |
|  | 18-30 yrs (235.58) | 51-65 yrs (235.24) | 0.978 | 0.001 | 470 |
|  | 18-30 yrs (202.33) | 66-75 yrs (228.76) | 0.069 | 0.089 | 411 |
|  | 18-30 yrs (195.72) | >75 yrs (221.93) | 0.102 | 0.082 | 396 |
|  | 31-50 yrs (169.83) | 51-65 yrs (174.76) | 0.606 | 0.028 | 342 |
|  | 31-50 yrs (137.47) | 66-75 yrs (159.96) | 0.030* | 0.129 | 283 |
|  | 31-50 yrs (131.08) | >75 yrs (152.88) | 0.049* | 0.120 | 268 |
|  | 51-65 yrs (83.29) | 66-75 yrs (94.54) | 0.107 | 0.122 | 173 |
|  | 51-65 yrs (76.69) | >75 yrs (87.26) | 0.136 | 0.119 | 158 |
|  | 66-75 yrs (49.92) | >75 yrs (50.11) | 0.971 | 0.004 | 99 |

HMPs-eT=Herbal Medicinal Products except Teas, HTs=Herbal Teas
