# Supplementary material for: Trends and Patterns for the Use of Herbal Medicinal Products for Gynaecological Ailments
Source: Phytother Res. 2026 Apr 6;40(6):3580–94. doi: 10.1002/ptr.70321 (PMC13254121; doi:10.1002/ptr.70321)
Supplement: Supplementary file 2 — Table S2: Frequencies of treatment with HMPs in relation to severity of symptoms. [file PTR-40-3580-s003.docx]

**Supplementary Table 2: Frequencies of Treatment with HMPs in Relation to Severity of Symptoms.**

|  | **Frequencies of HMP Application** | | | | | |
| --- | --- | --- | --- | --- | --- | --- |
| **Severity** | **Mens (n=222)** | | **Meno (n=301)** | | **uUTI (n=840)** | |
|  | **HMPs e.T.**  **(n=175)** | **HT**  **(n=47)** | **HMPs e.T.**  **(n=278)** | **HT**  **(n=23)** | **HMPs e.T.**  **(n=482)** | **HT**  **(n=358)** |
| **0** | 0.6%  (n=1) | 0 %  (n=0) | 0.7%  (n=2) | 14.3% (n=3) | 0.4%  (n=2) | 2.0% (n=7) |
| **1** | 6.3%  (n=11) | 8.7%  (n=4) | 5.5% (n=15) | 0%  (n=0) | 4.9% (n=23) | 8.0% (n=28) |
| **2** | 26.4%  (n=46) | 17.4%  (n=8) | 25.9% (n=71) | 23.8% (n=5) | 22.0% (n=104) | 25.8% (n=90) |
| **3** | 36.2%  (n=63) | 39.1%  (n=18) | 35.4% (n=97) | 28.6% (n=6) | 34.1% (n=161) | 30.9% (n=108) |
| **4** | 25.9%  (n=45) | 30.4%  (n=14) | 25.2% (n=69) | 23.8% (n=5) | 28.6% (n=135) | 25.6% (n=89) |
| **5** | 4.6%  (n=8) | 4.4%  (n=2) | 7.3% (n=20) | 9.5% (n=2) | 10.0% (n=47) | 7.7% (n=27) |
| no answer | n=1 | n=1 | n=4 | n=2 | n=10 | n=9 |

0 = ”no complaints”, 5 = ”strongest complaints”, 1, 2, 3 and 4 = any perceived severity in between “no complaints” and “strongest complaints”; Mens= menstrual complaints, Meno=menopausal complaints, uUTIs=uncomplicated urinary tract infections, HMPs e.T.=Herbal Medicinal Products except Teas, HTs=Herbal Teas
