# Supplementary material for: Trends and Patterns for the Use of Herbal Medicinal Products for Gynaecological Ailments
Source: Phytother Res. 2026 Apr 6;40(6):3580–94. doi: 10.1002/ptr.70321 (PMC13254121; doi:10.1002/ptr.70321)
Supplement: Supplementary file 3 — Table S3: Treatment habits of HMPs: are there significant differences in preferences for certain pharmaceutical forms (HMPs‐eT vs. HTs) and the frequency of use (‘daily’ vs. ‘if required’)? (chi‐squared‐test). [file PTR-40-3580-s002.docx]

**Supplementary Table 3: Treatment Habits of HMPs:** Are there significant differences in preferences for certain pharmaceutical forms (HMPs-eT versus HTs) and the frequency of use (“daily” vs. “if required”)? (Chi^2^-test)

| **Indication** | ***p*** | ***Cramer’s V*** | **Pearson Chi-Square *X*^2^** | **N** |
| --- | --- | --- | --- | --- |
| **Menstrual Complaints** | 0.003* | 0.212 | 9.715 | 216 |
| **Menopausal Complaints** | 1.547E-13* | 0.428 | 54.509 | 297 |
| **Uncomplicated Urinary  Tract Infections** | 1.3749E-7* | 0.184 | 27.758 | 823 |

HMPs e.T.=Herbal Medicinal Products except Teas, HTs=Herbal Teas
