# Supplementary material for: Trends and Patterns for the Use of Herbal Medicinal Products for Gynaecological Ailments
Source: Phytother Res. 2026 Apr 6;40(6):3580–94. doi: 10.1002/ptr.70321 (PMC13254121; doi:10.1002/ptr.70321)
Supplement: Supplementary file 4 — Table S4: Are there significant differences in preferences for certain pharmaceutical forms (HMPs‐eT vs. HTs) and the duration of use (‘short‐term use’ vs. ‘long‐term use’)? (chi‐squared‐test). [file PTR-40-3580-s004.docx]

**Supplementary Table 4:** Are there significant differences in preferences for certain pharmaceutical forms (HMPs-eT versus HTs) and the duration of use (“short-term use” vs. “long-term use”)? (Chi^2^-test)

| **Indication** | ***p*** | ***Cramer’s V*** | **Pearson Chi-Square *X*^2^** | **N** |
| --- | --- | --- | --- | --- |
| **Menstrual Complaints** | 3.8556E-11* | 0.449 | 43.686 | 217 |
| **Menopausal Complaints** | 9.1966E-7* | 0.286 | 24.089 | 295 |
| **Uncomplicated Urinary  Tract Infections** | 0.198 | 0.048 | 1.856 | 807 |

HMPs-eT.=Herbal Medicinal Products except Teas, HTs=Herbal Teas
