# Supplementary material for: Trends and Patterns for the Use of Herbal Medicinal Products for Gynaecological Ailments
Source: Phytother Res. 2026 Apr 6;40(6):3580–94. doi: 10.1002/ptr.70321 (PMC13254121; doi:10.1002/ptr.70321)
Supplement: Supplementary file 5 — Table S5: Perceived therapeutic effectiveness of HMPs: are there significant differences in preferences for certain pharmaceutical forms (HMPs e.T. vs. HTs) and the perceived therapeutic effectiveness (‘very good’, ‘moderate‐distinct’, ‘minimal‐mild’, ‘unchanged‐worsend’)? (Mann–Whitney U‐test). [file PTR-40-3580-s001.docx]

**Supplementary Table 5: Perceived Therapeutic Effectiveness of HMPs:** Are there significant differences in preferences for certain pharmaceutical forms (HMPs e.T. versus HTs) and the perceived therapeutic effectiveness (“very good”, “moderate-distinct”, “minimal-mild”, “unchanged-worsend”)? (Mann-Whitney U-test)

| **Indication** | **Group I**  **HMPs e.T.**  ***m*_Rang_** | **Group II**  **HT**  ***m*_Rang_** | ***p*** | ***r*** | **N** |
| --- | --- | --- | --- | --- | --- |
| **Menstrual Complaints** | 109.46 | 119.09 | 0.318 | 0.067 | 222 |
| **Menopausal Complaints** | 151.47 | 145.37 | 0.728 | 0.020 | 301 |
| **Uncomplicated Urinary Tract Infections** | 404.40 | 442.18 | 0.016* | 0.083 | 840 |

HMPs e.T.=Herbal Medicinal Products except Teas, HTs=Herbal Teas
