# Supplementary material for: Trends and Patterns for the Use of Herbal Medicinal Products for Gynaecological Ailments
Source: Phytother Res. 2026 Apr 6;40(6):3580–94. doi: 10.1002/ptr.70321 (PMC13254121; doi:10.1002/ptr.70321)
Supplement: Supplementary file 6 — Table S6: Perceived therapeutic effectiveness of specific drugs: are there significant differences in preferences for certain pharmaceutical forms (HMPs e.T. vs. HTs) and the perceived therapeutic effectiveness of specific drugs (‘very good’, ‘moderate‐distinct’, ‘minimal‐mild’, ‘unchanged‐worsend’)? (Mann–Whitney U‐test). [file PTR-40-3580-s005.docx]

**Supplementary Table 6: Perceived Therapeutic Effectiveness of Specific Drugs:** Are there significant differences in preferences for certain pharmaceutical forms (HMPs e.T. versus HTs) and the perceived therapeutic effectiveness of specific drugs (“very good”, “moderate-distinct”, “minimal-mild”, “unchanged-worsend”)? (Mann-Whitney U-test)

| **Indication** | **Group I**  **HMPs e.T.**  ***m*_Rang_** | **Group II**  **HT**  ***m*_Rang_** | ***p*** | ***r*** | **N** |
| --- | --- | --- | --- | --- | --- |
| ***Arctostaphylos uva-ursi* (L.) Spreng.** | 114.25 | 136.67 | 0.137 | 0.098 | 231 |
| ***Betula pendula* Roth,**  ***Arctostaphylos uva-ursi* (L.) Spreng.,**  ***Achillea millefolium* L.** | 163.79 | 166.80 | 0.854 | 0.010 | 332 |

HMPs e.T.=Herbal Medicinal Products except Teas, HTs=Herbal Teas
